# How identification with and attachment to place affects preference to move in later life: smallest space analysis

**DOI:** 10.3389/fpsyg.2025.1704089

**Published:** 2025-11-05

**Authors:** Stefan White, Stephen Walsh, Stephanie Shuttleworth, Neil Dagnall

**Affiliations:** School of Architecture / Health and Education, Manchester Metropolitan University, Manchester, United Kingdom

**Keywords:** smallest space analysis, ageing in place, Age-Friendly Environments, rightsizing, social identification

## Abstract

As populations age and urbanize, there is a need for housing and neighborhoods that support healthier, happier lives for older adults. While “Aging in Place” policies enable seniors to remain in their homes and communities, critics argue they overlook the complex physical, social, and psychological factors necessary for positive aging. Particularly, policies that focus on the dwelling or proximity to care and failure to address older adults’ holistic needs. Positive alternatives, such as the “Age-Friendly Environments,” proposed by the World Health Organization, emphasize public health interventions that create neighborhoods where older people maintain social connections and live in supportive environments, regardless of accommodation type. This research, drawing on UK Understanding Society survey data, utilized Smallest Space Analysis (SSA) to identify what influences older adults’ preferences to stay or move. Findings suggest that a significant predictor of housing choice was the neighborhood as a social “place.” Specifically, it is a location with which individuals identified, attached, and embedded. This outcome held more weight than individual attributes like house type, financial status, or social position. Results highlighted the need for further empirical investigation into the centrality of neighborhood identification in older adults’ housing decisions.

## 1 Introduction

This article reports on an interdisciplinary research program exploring theoretical and empirical gaps in the understanding of residential location and health equity in positive experiences of aging (i.e., Location and Equity in Ageing Positively, LEAP). LEAP combines architectural and psychological perspectives to assess the utility of a social identity approach (SIA) to equitable urban aging.

The global population is aging rapidly, and the proportion of people aged over 65 years will rise from around 10% in 2024 to over 16% by 2050 (Chesnaye et al., 2024). In the UK, the situation is more dramatic: at present, almost one in five UK residents is aged over 65, and this is projected to rise to one in four people by 2050 (Begde et al., 2024). Currently, many older adults live in homes that are not appropriate, or no longer appropriate, for their needs. Indeed, they remain in their current homes until a crisis, such as health deterioration or eviction forces them to move from their supportive social networks.

Additionally, older adults experience a shortage of diverse, accessible, and age-friendly housing in both existing communities and desired new locations. This deficit limits their ability to make active, positive choices about living arrangements since desired options are not available. This is a defining characteristic of the “rightsizing gap” (Hammond et al., 2018).

Evidence shows that the majority of older people do wish to remain in their existing home, and that this percentage increases as they age alongside a corresponding increase in satisfaction with home and neighborhood (Hillcoat-Nallétamby and Ogg, 2014). A broadly held conclusion is that this is attributable to older people’s sense of attachment to their local environment, providing a familiar landscape helping them to maintain a sense of competence in going about their daily activities (Lawton, 1989). Consequently, with very few older people moving every year (circ 3.5%) in the UK, “Ageing in place” has become the predominant concept framing housing policy and practice (Forsyth and Molinsky, 2021), emphasizing helping older people to remain in their home as they age (Boaz et al., 1999; Carter and Hillcoat-Nalletamby, 2015). However, it is clear that many older people live in housing, which becomes increasingly inappropriate as they grow older, with data indicating that up to 4Million older people in the UK potentially wish to move (Hammond et al., 2018).

Critically, theorists (e.g., Hillcoat-Nallétamby and Ogg, 2014; Yarker et al., 2023) observe that while advocates assume “Ageing in Place” is beneficial to older people, it fails to account for the relational nature of place and the active role of older people play in generating a local environment with others. Their research shows satisfaction may be more shaped by “a desire to attach to people” than a specific domicile (Hillcoat-Nallétamby and Ogg, 2014).

Research into residential satisfaction in housing choices for older people highlights the complexity and interconnectedness between a range of aspects of the residential environment beyond the home (Johnson-Carroll et al., 1995), including interior and exterior features of housing and neighborhood, affecting mobility (Fernández et al., 2003; Kahana et al., 2003); systemic features of infrastructure and services (Phillips et al., 2004); and social relationships within the neighborhood (Oh, 2003; Fong et al., 2021). Inter-personal research shows that the “person–environment interaction” involves “older people” balancing a wide range of personal factors in relation to the particular place they live when actually deciding to move or stay in a process described as “Option recognition” (Peace et al., 2011).

This research observed that “Ageing in Place” broadly fails to address the material circumstances of different groups of older people in relation to their “obtainable” housing options; does not account for the pragmatic acceptance as satisfactory of undesirable circumstances considered unchangeable; and has yet to fully account for the relational nature of attachment to place with respect to simultaneous, physical, psychological, and social factors (Yarker et al., 2023). These inadequacies require urgent attention as the role of a place in addressing health inequality for older adults is highlighted by the World Health Organization’s (2007) targeted Age-friendly Cities and Communities programs, which emphasize the involvement of older residents in the consideration and improvement in local responses to the determinants of healthy ageing in relation to eight interlocking domains, such as housing, outdoor space and buildings, communication and information, social respect and participation, civic participation and employment, transport, and community health and support.

In particular, it is important to improve understanding of the nature of place-focused interventions (location), which could support improved experiences for older residents (ageing positively), who are most likely to have reduced healthy life expectancy due to structural and social health inequalities (equity), as they may have a reduced capacity to effect change in features of their neighborhood environment without additional support.

In undertaking the present research, we intended to establish whether it was possible to identify factors, or variables, amongst a cohort of older adults, which were indicative of who wants to stay in their current homes and who wants to move. An important theoretical lens that offers a means of distinguishing between where people live, their neighborhood, as a physical entity (i.e., as a defined geographical area of co-located residents) and as a psychological entity (i.e., as a group of people who share a sense of themselves as residents of a given neighborhood) is the social identity approach (Jetten et al., 2017).

We explored a large data set with variables related to place identification, neighborhood social connections, and local resources in order to understand if it is possible to identify specific features that could indicate a preference to stay or move for older people. This research supports efforts to better define the nature of the “attachment” that older people form with their place of residence by exploring the relationship between preference to stay or move, and variables that could indicate the relationship between a social identity approach and critical psychological, physical, and personal dimensions of place attachment (PPP—Scannell and Gifford, 2010).

Attachment is a commonly ascribed mechanism by which specific residential locations come to hold sufficient meaning to determine the preference of older people who wish to stay (Lawton, 1989). Peng et al. (2020) note that the conception of self was introduced by Proshansky et al. (2014) in his definition of “place identity”, but argue there remains an ambiguity about the relationship between the identity of a place and the identification of individuals and groups with that place compounded by a range of related but distinct terms, such as place attachment, belongingness, rootedness, sense of place, place dependence, and place satisfaction (e.g., identity process theory, Twigger-Ross and Uzzell, 1996; Lewicka, 2011). Despite a significant increase in publications since 2006 (Peng et al., 2020), advances in the theoretical and empirical aspects of understanding “the bonds between humans and places” (Hernández et al., 2020) require further clarification.

Lewicka (2011) advocates an attempt at reconciliation by Scannell and Gifford (2010), who posit a triadic person, psychological process, and place (PPP) model. This provides an inclusive view of place attachment that structures a range of existing definitions. They argue that the PPP model is compatible with quantitative and qualitative studies and avoids the limitations of many previous models (Scannell and Gifford, 2010). Accordingly, the PPP approach is linked to a variety of environmental sociology and psychology researchers (e.g., Manzo and Devine-Wright, 2013). The PPP model identifies three psychological processes in the generation of place attachment: affect (emotion), cognition (identity), and behavior (action). The Person dimension forms place attachment at individual and group levels. The Place dimension then subdivides into social and physical place attachment (Scannell and Gifford, 2010).

Using the PPP model, Maricchiolo et al. (2021) argue that a localized place-based social identity influences well-being through three key components of place attachment: place identity, social relations, and lack of resources. Examining this, Maricchiolo et al. (2021) found that individual happiness and wellbeing were correlated with local social identity, and that place and social identity (positively) and lack of resources (negatively) mediated the relationship. We highlight that the components of attachment identified by Maricchiolo show strong alignment with the overall basket of variables tested in the preceding analysis.

In what follows, we borrow an argument from Haslam et al. (2024) and argue that the unique value of the social identity approach (SIA) to understanding the psychology of neighborhood identification and belongingness is that SIA specifies the social identity processes that shape both group cohesion and perceptions of environmental quality. SIA offers a means of not only distinguishing between where people live, their neighborhood, as a physical entity (i.e., as a defined geographical area of co-located residents) and as a psychological entity (i.e., as a group of people who share a sense of themselves as residents of a given neighborhood) but also enables a connection to health (Haslam et al., 2020). The SIA to wellbeing (Jetten et al., 2017; Haslam et al., 2018) suggests that the subjective sense of belonging created through meaningful social identification is a central psychological process for the generation of wellbeing (Sani, 2012; Junker et al., 2019).

This specification, in turn, may be useful in understanding why it is that some older people want to stay in their present accommodation and some want to move. It may be that older people’s housing aspirations and “rightsizing” are a function of psychology as well as geography and practicality. Understanding these psychological and social dimensions is crucial for developing a comprehensive view of older adults’ preference to stay or move home or neighborhood.

Smallest Space Analysis (SSA) is a powerful statistical technique used to identify and visualize the relationships between multiple variables by representing them in a spatial map. This method is particularly useful in the context of understanding the role of place in older adults’ preferences to stay or move, as it allows researchers to distil complex data into an easily interpretable format. SSA plots conceptual proximity or associations between factors in a multidimensional space, positioning closely related items nearer to each other and more distantly related items farther apart, revealing underlying patterns in the data (Weisman Openhaim et al., 2024).

In this study, SSA was employed to analyse data from the UK Household Longitudinal Study, focusing on individuals over 55 years of age. By examining variables, including employment status, social support, neighborhood cohesion, and perceived safety, SSA helped identify key factors that influence whether older adults prefer to stay in their current homes or move. This approach is particularly valuable because it highlights the multidimensional nature of housing preferences, encompassing physical, social, and psychological dimensions.

The applicability of SSA in this area lies in its ability to uncover hidden patterns and relationships within large datasets, providing insights that might not be apparent through traditional analysis methods. By visualizing these relationships, SSA facilitates a deeper understanding of the complex interplay between factors that contribute significantly to the preference to stay or move from a house or neighborhood. This information is important as it can inform the development of effective housing policies and interventions for older adults, which address issues, such as the rightsizing gap (Hammond et al., 2018).

## 2 Method

Multidimensional scaling (MDS) is a statistical method used to measure and visualize how similar or different a group of items is. It works by taking data about similarities—such as ratings or patterns of confusion between items—and creating a map that shows their relationships. On this map, an analysis places items more alike closer together, while those that are less alike are farther apart. The items can be physical things we sense or ideas we think about. By looking at the layout of the map, researchers can figure out the key features that explain the similarities and differences, or check if their initial ideas about the data were correct (Hout et al., 2013, p.93).

This study used a particular type of MDS, smallest space analysis (SSA), to identify conditions of place and home that influence older persons’ preferences to move (i.e., those who answer “yes” to prefer to move) or stay (i.e., those who answered “agree” or “strongly agree” to plan to stay in the neighborhood). SSA is a manifestation of Multi-Dimensional Scaling (MDS; e.g., Raveh and Tapiero, 1980), a statistical method that reduces very large amounts of data into a manageable size, thereby enabling researchers to make inferences and form conclusions (Jaworska and Chupetlovska-Anastasova, 2009). The analytic approach/technique attains this by condensing data into a straightforward spatial map, which allows for the identification and illustration of important relationships in the most economical manner (Mugavin, 2008).

The data set was from Wave 6 of the Understanding Society (UK Household Longitudinal Study) data set, University of Essex/UK Data Service (2024), containing 45,433 responses, and, in our analysis, we focused on data from individuals >55 years of age. When “not sure” and answers other than those categorized as “prefer to stay” or “prefer to move,” as well as duplicate data (i.e., those who answered positively to both prefer to stay and prefer to move) were removed, 1782 individuals remained in the “prefer to move” category, and 9,825 in the “prefer to stay category.” Any missing data were not substituted. Manchester Metropolitan University (MMU EthOS no. 26319) granted ethical approval for the secondary data analysis undertaken in this project.

### 2.1 Analysis

The goal when using MDS is to identify dimensions affecting perception or behavior. In this instance, dimensions that impact the decision to move house (or remain *in situ*) may not have been readily evident in the available data. This gives the analyst an overview of the relationships between variables.

These insights are valuable in psychologically oriented research of the present type, which seeks to draw inferences from data derived from scaling, sorting, or ranking tasks as well as from questionnaires (Woosley et al., 2004). MDS is an excellent data mining technique. The method relies on binary data and a representation of relationships between “things” to produce structure. The closer these objects are, the stronger the relationships. Regional interpretation of each SSA plot identified a clear region in which the factors pertinent to both stay and move clustered around the respective preference. With regard to stability, Tucker’s coefficient of congruence is representative of how well the spatial distribution within the SSA represents the actual co-occurrence of variables. Tucker’s coefficient of congruence was 0.94 for “stayers” and 0.94 for “movers.” According to Lorenzo-Seva and Ten Berge (2006), when Tucker’s congruence coefficient is used to assess the similarity of factor interpretations, it is desirable to have a critical congruence level less than unity that can be regarded as indicative of identity of the factors. Lorenzo-Seva and Ten Berge’s (2006) results suggest that a value in the range 0.85–0.94 corresponds to a fair similarity, while a value higher than 0.95 implies that the two factors or components compared can be considered equal.

In this context, the purpose of the MDS analysis was to identify variables in the physical, social, or economic environment of the home or neighborhood that subsequent studies should investigate. The analytic process followed an iterative process. This began with a consideration of 306 variables in a data set that included 45,433 individual cases. Of these variables, on the basis of the author’s judgement, 62 were suitable for coherent dichotomization. For these, the authors calculated the Jaccard’s coefficient, which is a measure of association without which SSA cannot proceed. Next, based on respondents who preferred to stay in their current residence (*n* = 28,671) (vs. move, 12,679), the data were split into two files. This included removal of other data (e.g., “do not know”). Following initial reviewer feedback, no variables were removed on the basis of occurrence frequency. SPSS (Proxscal) was then used to calculate proximities between variables and present these in a two-dimensional space.

Following dichotomization conducted on the basis of “yes” or “no,” the variables are listed below in the truncated format as included in the analysis:

**Table 1:** 

1. Children
2. lived_fEver (always lived there)
3. PrefToMove
4. PrefToStay
5. Employed
6. Retired
7. Married
8. Car_or_Van
9. UK_born
10. Edn
11. Attitude_local_med_services
12. Attitude_local_shopping
13. Attitude_local_leisure
14. CloseKnitNeighborhood
15. PerceivedSupport
16. PerceivedTrust
17. FearOfCrime
18. FriendsSameAge
19. FriendsSameRace
20. FriendsSameEd
21. FriendsWithJob
22. FriendsSimilarIncome
23. FriendsInArea
24. FriendswhoareFamily
25. SocialWebsite
26. VisitFriends
27. TooBusyFriends
28. FinancialReasons
29. HealthIllnessDisability
30. NoPublicTransport
31. PoorPublicTransport
32. CantAccesPTransport
33. NoAccesstoCar
34. FearCrime (Different in these surveys to fear of crime)
35. CaringResponsibilities
36. Memberoflistedorg
37. Longillordiab
38. Carer
39. Livedconstant
40. Workloc
41. Employerpension
42. Haspension
43. Serps (State Earnings Related Pension Scheme)
44. Incapacity
45. Employment_allowance
46. Disability
47. CarersAllow
48. DisabLivingAllow
49. PersIndPayment
50. AttAll (attendance allowance)
51. IndInjuryBen
52. AnyOtherBenefitDisability
53. NoOtherBenefit
54. CurrentFinSit
55. FutureFinSit
56. RegularSaver
57. PrivatePension
58. Expectworkpostretirement
59. Adequacyexpctretirincome
60. Belong
61. LocalFriends
62. OwnHome

(Note: In the Understanding Society survey, “fear crime” and “fear of crime” are discrete variables)

Within MDS/SSA, indirect variables with a strong effect in relation to the preference to stay or move designate dimensions of place attachment. The graph below illustrates the results.

## 3 Results

The normalized raw stress values for both datasets are as follows: stay group, 0.10764; and move group, 0.11065. These values are well below the commonly accepted threshold of 0.15, which suggests that a two-dimensional solution provides a good fit to the data. Adding a third dimension would likely result in diminishing returns in terms of improved fit.

**Figure 1:**
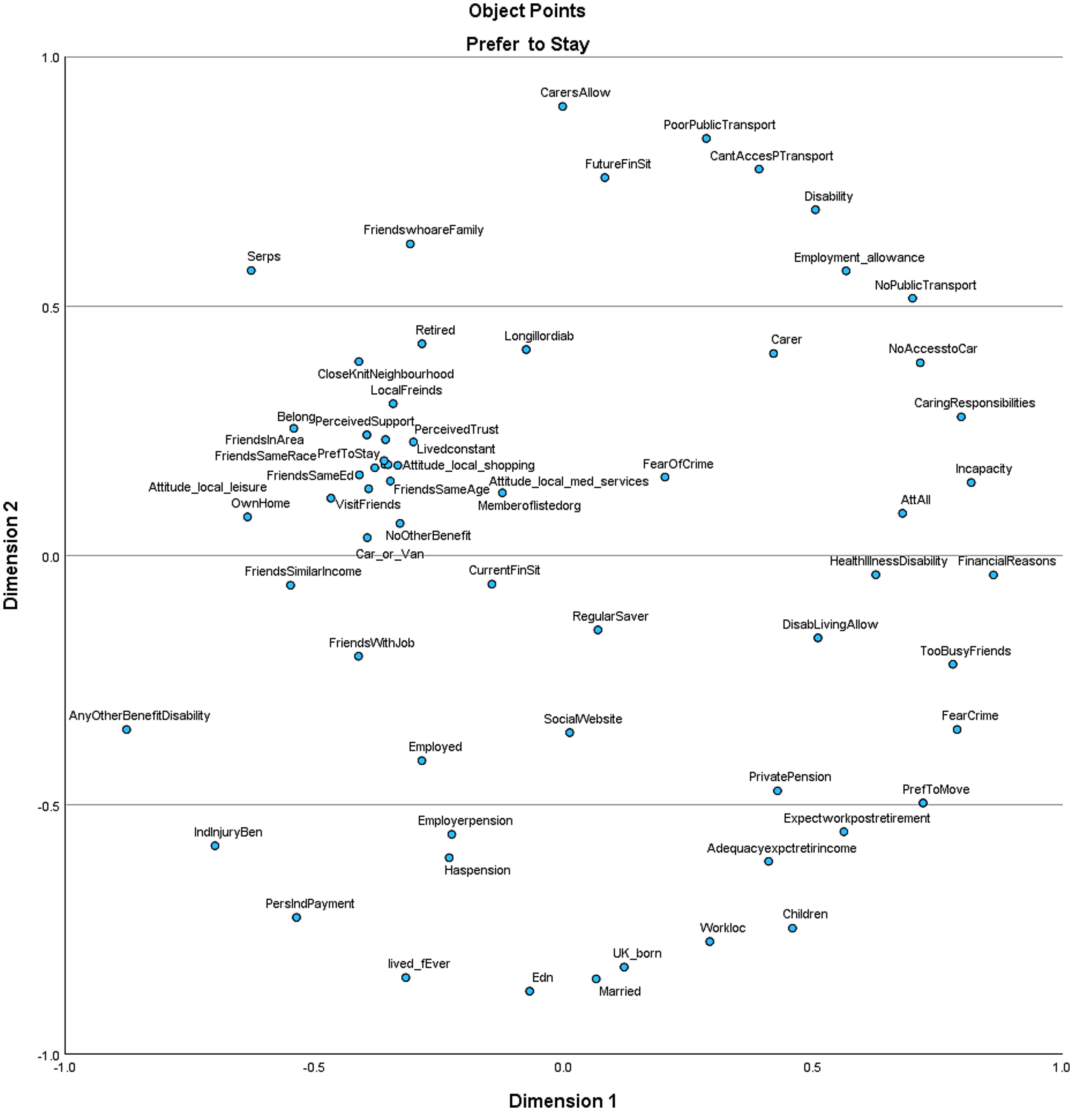
Factors influencing preference to move.

**Figure 2:**
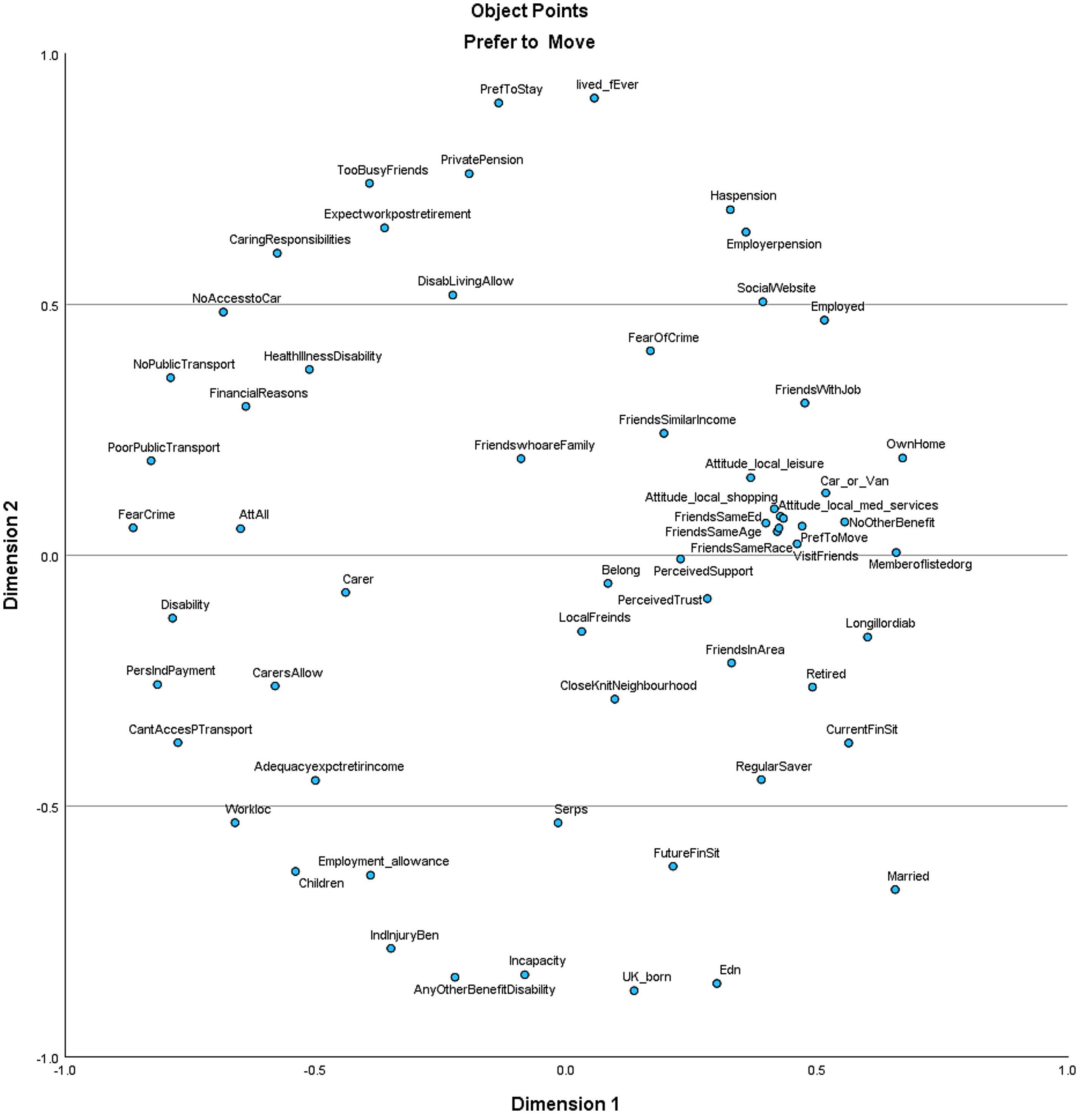
Factors influencing preference to stay.

The analysis presented here shows that when considered in terms of the variables most clustered around either preference to stay or preference to move, in the present model, psychological, physical, and personal (PPP) factors come to the fore. These include *Social relations and networks* [e.g., Attitudes re local facilities]; *Social and physical neighborhood resources* [e.g., friends, members of one of the listed organizations]; and *identification with the place* [e.g., perceived support]. Furthermore, the vertical axis on each plot can be interpreted as “important to one’s preference to stay or move” [higher on the plot = more important], and the horizontal axis can be understood as either “have or do not have” [left to right] (see Figures 1, 2).

## 4 Discussion

The results suggest that people who would prefer not to move identify more with and are more embedded in their communities and localities than those who would prefer to move, consistent with embeddedness and neighborhood identification, as per Haslam et al. (2018). In this context, identification means sharing a sense of “us” with others in their neighborhood.

These findings, illustrating that a basket of factors come together in a way suggestive of belongingness, are interesting, in part, because they are consistent with research that has considered workplace turnover and which shows that, when understood through a social identity theory lens, peripheral positions in informal networks (solidarity ties and instrumental ties) of a marginal social identity in the workplace is related to higher turnover intention (Kwon, 2017).

Social identity theory is, nowadays, most often considered as part of the social identity approach. The social identity approach is a psychological metatheory incorporating social identity theory (SIT) and self-categorization theory (SCT) (Haslam et al., 2020). The social identity paradigm is prominent in social psychology since it approaches analysis from a unique position. Rather than starting with “the individual in the group,” it proceeds from the understanding that one must begin with a consideration of how the group influences the individual (Reicher et al., 2010).

Tajfel and Turner (1979) defined social identity as an individual’s knowledge that they belong to certain social groups and that membership of these groups has an emotional value and is significant to them. It focuses on the “we’s” people ascribe to and how, when “we” self-categorize as a group member, and “we” interact with “others.” SCT has a focus on the shift behind people’s self-categorization from idiosyncratic individuals to individuals as members of collective groups. It is interesting how we behave and how others behave toward us based on our group memberships or social identities.

Bringing a social identity approach lens to bear on an analysis of older adults’ housing preferences is important and consequential. In a recent paper, Haslam et al. (2024) report that prevailing models tend to neglect the fact that a neighborhood is a spatially defined social grouping of people that is potentially internalized into a person’s sense of self (for example, as residents of Manchester). Moreover, individuals internalize their spatially defined social groups in ways that have a demonstrable impact on their lives and wellbeing (Haslam et al., 2024). In particular, in their consideration of neighborhood social identities (not explicitly measured in the data used herein) and mental health, Haslam et al. (2024) suggest that neighborhood is a psychological rather than a geographic entity.

Moreover, and flowing directly from this insight that neighborhood is a psychological, social identification, built on neighborhood belongingness, predicts that when residents share a sense of common neighborhood identity, this will tend to increase the likelihood that they see their neighborhood as cohesive and will therefore increase their willingness to work together. Intuitively, this observation by Haslam et al. (2024) makes good sense.

Furthermore, this (evidenced) recognition that shared identification increases neighborhood cohesiveness and relates to positive mental health sits easily with the observation presented herein that the more embedded people are in their neighborhoods, the less likely they are to want to move. It may well be that the “rightsizing gap” (Hammond et al., 2018) is as much about neighborhood identity and the psychology of neighborhood belongingness, identification, and connectivity as it is about domestic square footage, wet rooms, or the presence of steps. The results presented here are consistent with this inference. However, neighborhood social identification was not a variable analyzed—rather social identity is a conceptual tool that is rationally and coherently applied to make sense of current data. To empirically test the centrality of neighborhood identification, investigators need to undertake further research.

The concept of neighborhood identification, as highlighted by Haslam et al. (2024), underscores the importance of social identity in shaping housing preferences. Older adults who feel an intense sense of belonging and identification with their neighborhood are more likely to prefer staying in their current homes. This finding aligns with social identity theory, which posits that individuals derive a sense of self from their membership in social groups (Tajfel and Turner, 1979). The embeddedness in local communities fosters a sense of security, support, and continuity, which are vital for the wellbeing of older adults.

The results presented herein are consistent with previous research that has explored the relationship between social identity and housing preferences. For instance, Mulliner et al. (2020) found that the desire to maintain social connections and a sense of community influenced older adults’ housing choices. Similarly, research by Wister (2005) highlighted the role of the built environment in supporting health and longevity. However, this study adds a new dimension by using smallest space analysis (SSA) to uncover the complex interplay between numerous factors, which in turn are suggestive of embeddedness, neighborhood identification, and integration in one’s community as key factors that foster a sense of contentment with place in this cohort of older adults.

Understanding of the nature of aging in a place, as the formation of an attachment created by a familiar landscape which supports competence (Lawton, 1989), does not appear to be sufficient to accommodate the wide range of different familial, geographical, and environmental contexts of diverse groups of older people. The large representative UK data sample analyzed here is therefore suggestive that place attachment, which defines the preference of older people to stay or move, may comprise a broader set of features. In our analysis, there is a tentative alignment in the basket of variables in our data space and the three components of place attachment set out by Scannell and Gifford (2010) in the PPP model and investigated by Maricchiolo et al. (2021) in relation to features key to “influencing well-being.” For discussion, we categorize them as:

*Social relations and networks* [Has pension / No Disability Benefit / No Other Benefit / Employer pension / Current Financial Situation / Attitude local leisure facilities]; *Social and physical neighborhood resources* [Friends Similar Income / Friends With Job / Friends In Area / Friends who are Family / Social Website / Visit Friends / Member of listed org / Local Friends]; and *identification with the place* [Fear Of Crime / Close Knit Neighborhood / Perceived Support / Perceived Trust / Feeling of belonging].

### 4.1 Limitations

Despite the preliminary insights it has generated, the study has limitations. The use of cross-sectional, secondary data from the Understanding Society Survey may not capture all nuances of older adults’ housing preferences; the measures included are indicative of identification and belonging but are not explicit measures. The focus on individuals over 55 years of age may not fully represent or capture the diversity within the older adult population. Future research should include quantitative measures that can directly assess dimensions of interest; qualitative methods to gain a deeper understanding of the factors influencing the housing choices of older adults; and explore the role of neighborhood social identification in greater detail. Empirical studies that examine how neighborhood identity influences housing preferences can provide more robust evidence to support the findings of this study. Additionally, research should investigate the impact of distinct types of housing interventions on older adults’ wellbeing. For example, studies could examine the effectiveness of community-based programs that promote social engagement and support.

### 4.2 Conclusion

The SSA illustrated that those in peripheral relationships with their neighborhood wish to move, whereas those interdependently connected or embedded in their neighborhoods are less likely to want to move. These findings indicate that older people consider a range of factors concurrently when they are considering their preference to stay or move home and neighborhood in later life.

The research therefore supports the argument that to address the rightsizing gap and create “Age-friendly” environments, theorists and policymakers should consider the neighborhood holistically as a social “place” which residents wish to identify, attach, or become embedded within, rather than individual features such as a house type, financial status, or social position.

## Data Availability

Publicly available datasets were analyzed in this study. This data can be found here: The data set was from Wave 6 of the Understanding Society (UK Household Longitudinal Study) data set University of Essex/UK Data Service (2024) containing 45,433 responses and, in our analysis, we focused on data from individuals >55 years of age, 12.022 of whom had a preference to remain in their current housing; and 3,211 who would prefer to move.

## References

[ref1] Begde A. Jain M. Goodwin M. Brayne C. Barnes L. Brooks R.. (2024). Exploring factors influencing willingness of older adults to use assistive technologies: evidence from the cognitive function and ageing study II. Inf. Commun. Soc. 27, 368–385. doi: 10.1080/1369118x.2023.2205915

[ref2] Boaz A. Hayden C. Miriam B. (1999). Attitudes and aspirations of older people: a review of the literature. Report 101: United Kingdom Government Department of Social Security. Leeds: Corporate Document services.

[ref39] Carter L. Hillcoat-Nalletamby S. (2015). Housing for Older People in Wales: An Evidence review. Public Policy Institute for Wales. Cardiff.

[ref3] Chesnaye N. C. Ortiz A. Zoccali C. Stel V. S. Jager K. J. (2024). The impact of population ageing on the burden of chronic kidney disease. Nat. Rev. Nephrol. 20, 569–585. doi: 10.1038/s41581-024-00863-9, PMID: 39025992

[ref4] Fernández G. F.-M. Pérez F. R. Abuín J. M. R. (2003). Components of the residential environment and socio-demographic characteristics of the elderly. J. Hous. Elderly 18, 25–49. doi: 10.1300/J081v18n01_03

[ref5] Fong P. Cruwys T. Robinson S. L. Haslam S. A. Haslam C. Mance P. L.. (2021). Evidence that loneliness can be reduced by a whole-of-community intervention to increase neighbourhood identification. Soc. Sci. Med. 277:113909. doi: 10.1016/j.socscimed.2021.113909, PMID: 33866082

[ref6] Forsyth A. Molinsky J. (2021). What is aging in place? Confusions and contradictions. Hous. Policy Debate 31, 181–196. doi: 10.1080/10511482.2020.1793795

[ref8] Hammond M. Walsh R. White S. (2018). RIGHTSIZING: reframing the housing offer for older people. Greater Manchester combined authority (GMCA)/PHASE @ Manchester School of Architecture. Available online at: https://www.housinglin.org.uk/_assets/Resources/Housing/OtherOrganisation/Rightsizing_MSA_Final.pdf (Accessed October 27, 2025).

[ref9] Haslam S. A. Fong P. Haslam C. Cruwys T. (2024). Connecting to community: a social identity approach to neighborhood mental health. Personal. Soc. Psychol. Rev. 28, 251–275. doi: 10.1177/10888683231216136, PMID: 38146705 PMC11193917

[ref10] Haslam C. Jetten J. Cruwys T. Dingle G. A. Haslam A. S. (2018). The new psychology of health: unlocking the social cure. London: Routledge.

[ref11] Haslam S. A. Reicher S. Platow M. (2020). The new psychology of leadership: identity, influence and power. Second Edn London: Routledge.

[ref12] Hernández B. Hidalgo M. C. Ruiz C. (2020). “Theoretical and methodological aspects of research on place attachment” in Place attachment (London: Routledge), 94–110.

[ref13] Hillcoat-Nallétamby S. Ogg J. I. M. (2014). Moving beyond ‘ageing in place’: older people’s dislikes about their home and neighbourhood environments as a motive for wishing to move. Ageing Soc. 34, 1771–1796. doi: 10.1017/S0144686X13000482

[ref14] Hout M. C. Papesh M. H. Goldinger S. D. (2013). Multidimensional scaling. WIREs Cogn. Sci. 4, 93–103. doi: 10.1002/wcs.1203, PMID: 23359318 PMC3555222

[ref15] Jaworska N. Chupetlovska-Anastasova A. (2009). A review of multidimensional scaling (MDS) and its utility in various psychological domains. Tutor. Quant. Methods Psychol. 5, 1–10. doi: 10.20982/tqmp.05.1.p001

[ref16] Jetten J. Haslam S. A. Cruwys T. Greenaway K. H. Haslam C. Steffens N. K. (2017). Advancing the social identity approach to health and well-being: progressing the social cure research agenda. Eur. J. Soc. Psychol. 47, 789–802. doi: 10.1002/ejsp.2333

[ref17] Johnson-Carroll K. J. Brandt J. A. McFadden J. R. (1995). Factors that influence pre-retirees' propensity to move at retirement. J. Hous. Elderly 11, 85–105. doi: 10.1300/J081V11N02_06

[ref18] Junker N. M. van Dick R. Avanzi L. Häusser J. A. Mojzisch A. (2019). Exploring the mechanisms underlying the social identity–ill-health link: longitudinal and experimental evidence. Br. J. Soc. Psychol. 58, 991–1007. doi: 10.1111/bjso.12308, PMID: 30561049

[ref19] Kahana E. Kahana B. Kercher K. (2003). Emerging lifestyles and proactive options for successful ageing. Ageing Int. 28, 155–180. doi: 10.1007/s12126-003-1022-8

[ref20] Kwon H. W. (2017). A social embeddedness perspective on turnover intention: the role of informal networks and social identity evidence from South Korea. Public Pers. Manage. 46, 263–287. doi: 10.1177/0091026017717459

[ref21] Lawton M. P. (1989). Three functions of the residential environment. J. Hous. Elderly 5, 35–50. doi: 10.1300/J081V05N01_04

[ref22] Lewicka M. (2011). Place attachment: how far have we come in the last 40 years? J. Environ. Psychol. 31, 207–230. doi: 10.1016/j.jenvp.2010.10.001

[ref23] Lorenzo-Seva U. Ten Berge J. M. (2006). Tucker’s congruence coefficient as a meaningful index of factor similarity. Methodology 2, 57–64. doi: 10.1027/1614-2241.2.2.57

[ref24] Manzo L. Devine-Wright P. (2013). Place attachment: advances in theory, methods and applications. 1st Edn. London: Routledge.

[ref25] Maricchiolo F. Mosca O. Paolini D. Fornara F. (2021). The mediating role of place attachment dimensions in the relationship between local social identity and well-being. Front. Psychol. 12:645648. doi: 10.3389/fpsyg.2021.645648, PMID: 34421706 PMC8378270

[ref26] Mugavin M. E. (2008). Multidimensional scaling: a brief overview. Nurs. Res. 57, 64–68. doi: 10.1097/01.NNR.0000280659.88760.7c, PMID: 18091294

[ref1001] Mulliner E. Riley M. Maliene V. (2020). Older People’s Preferences for Housing and Environment Characteristics. Sustainability. 12:5723. doi: 10.3390/su12145723

[ref27] Oh J.-H. (2003). Social bonds and the migration intentions of elderly urban residents: the mediating effect of residential satisfaction. Popul. Res. Policy Rev. 22, 127–146. doi: 10.1023/A:1025067623305

[ref28] Peace S. Holland C. Kellaher L. (2011). Option recognition' in later life: variations in ageing in place. Ageing Soc. 31, 734–757. doi: 10.1017/S0144686X10001157

[ref29] Peng J. Strijker D. Wu Q. (2020). Place identity: how far have we come in exploring its meanings? Front. Psychol. 11:294. doi: 10.3389/fpsyg.2020.00294, PMID: 32218753 PMC7078666

[ref30] Phillips D. R. Siu O.-L. Yeh A. G. Cheng K. H. (2004). Factors influencing older persons' residential satisfaction in big and densely populated cities in Asia: a case study in Hong Kong. Ageing Int. 29, 46–70. doi: 10.1007/s12126-004-1009-0

[ref31] Proshansky H. M. Fabian A. K. Kaminoff R. (2014). “Place-identity: physical world socialization of the self (1983)” in The people, place, and space reader (London: Routledge), 77–81.

[ref32] Raveh A. Tapiero C. (1980). Finding common seasonal patterns among time series: an MDS approach. J. Econom. 12, 353–363. doi: 10.1016/0304-4076(80)90061-5

[ref33] Reicher S. Spears R. Haslam S. (2010). “The social identity approach in social psychology” in The social identity approach in social psychology. eds. Wetherell M. Mohanty C. T. (45–62.). (London: SAGE Publications Ltd),

[ref34] Sani F. (2012). “Group identification social relationships and health” in The social cure (New York: Psychology Press), 21–37.

[ref35] Scannell L. Gifford R. (2010). Defining place attachment: a tripartite organizing framework. J. Environ. Psychol. 30, 1–10. doi: 10.1016/j.jenvp.2009.09.006

[ref36] Tajfel H. Turner J. C. (1979). “An integrative theory of intergroup conflict” in The social psychology of intergroup relations. eds. Austin W. G. Worchel S. (Monterey, CA: Brooks/Cole), 33–47.

[ref37] Twigger-Ross C. L. Uzzell D. L. (1996). Place and identity processes. J. Environ. Psychol. 16, 205–220. doi: 10.1006/jevp.1996.0017

[ref38] Weisman Openhaim E. Amram Y. Glicksohn J. (2024). Empathy and the dark triad. J. Individ. Differ. 45, 176–184. doi: 10.1027/1614-0001/a000422

[ref40] Wister A. V. (2005). The built environment, health, and longevity. J. Hous. Elderly 19, 49–70. doi: 10.1300/j081v19n02_04

[ref41] Woosley S. A. Hyman R. E. Graunke S. S. (2004). Q sort and student affairs: a viable partnership? J. Coll. Stud. Dev. 45, 231–242. doi: 10.1353/csd.2004.0031

[ref42] World Health Organization (2007). Global age-friendly cities: a guide. Geneva, Switzerland: World Health Organization.

[ref43] Yarker S. Doran P. Buffel T. (2023). Theorizing “place” in aging in place: the need for territorial and relational perspectives. Gerontologist 64, 1–6. doi: 10.1093/geront/gnad002 PMC10809216 36655690

